# Does health service funding go where the need is? A prototype spatial access analysis for new urban contracts data

**DOI:** 10.1186/s12913-021-07370-8

**Published:** 2022-01-09

**Authors:** Julia Koschinsky, Nicole P. Marwell, Raed Mansour

**Affiliations:** 1 Center for Spatial Data Science, University of Chicago, 1155 E 60th St, Chicago, IL 60637 USA; 2 Crown Family School of Social Work, Policy, and Practice, University of Chicago, 969 E 60th St, Chicago, IL 60637 USA; 3 Chicago Department of Public Health, 333 S State St #200, Chicago, IL 60604 USA

**Keywords:** Health services, Administrative contracts data, Spatial access

## Abstract

**Background:**

Much of spatial access research measures the proximity to health service locations. We advance this research by focusing on whether health service funding is within walkable reach of neighborhoods with high hardship. This is made possible by a new administrative data source: financial contracts data for those human services that are delivered by nonprofits under contract with the government.

**Methods:**

In a prototypical spatial access study we apply a classic 2-step floating area catchment model for walkable network access to analyze 2018 data about contracted nonprofit health services funded by the Chicago Department of Public Health (CDPH). CDPH collected the data for the purpose of this study.

**Results:**

We find that the common container approach of aggregating contract amounts by provider headquarter locations in a given area (ignoring satellite service sites) underestimates the share of funding that goes to Chicago neighborhoods with higher hardship. Once service sites and spatial access are taken into account, a larger share of CDPH funds was found to be within walkable reach of Chicago’s high hardship areas. This was followed by low hardship areas (which could be driven by more headquarter locations there that do serve areas throughout the city). Medium hardship areas trail both, perhaps warranting closer attention. We explore these results by program type and neighborhood with a spatial decision support system developed for the health department.

**Conclusions:**

The typical approach for analyzing human service contracts based on headquarters is misleading -- in fact, we find that results are reversed when service sites and walkable access are taken into account. This prototype provides an alternative framework for avoiding these misleading results.

**Supplementary Information:**

The online version contains supplementary material available at 10.1186/s12913-021-07370-8.

## Introduction

Each year, government spends more than a trillion dollars [1, 2] in combined federal, state, and local funds to support hundreds of thousands of local service providers in a highly decentralized system of service provision in the U.S. Each city, county, and state manages its own system of allocating contracts to human service providers to meet resident needs [3, 4]. Government and its private sector partners make choices every day about where to make services available, affecting which city residents are best able to access these services. Even though the equitable allocation of public facilities like parks or libraries within jurisdictions is a classic planning problem, spatial access to contracted health and human services by funding amount has received less attention. In fact, the literature on contracting in human services and on the spatial accessibility of health services developed rather separately. Hence, a research gap exists in intersecting service contracting and spatial access research. With this article and previous related work [4], we are starting to close this gap by providing a prototype analysis of how the spatial distribution of contract funding for such services can be related to the spatial concentration of need in urban neighborhoods of the City of Chicago.

Specifically, we address the questions: (1) are publicly funded but privately provided health services delivered where needs are concentrated? and (2) which locations lack spatial access to such human services and to per capita funding for them? To address these questions, we conduct a spatial accessibility analysis with an underutilized administrative data source: data on government financial contracts with private nonprofit service providers. Our initial scope included human services across all human service departments in all non-federal jurisdictions (city, county and state). Due to challenges with open data described elsewhere [5], we narrowed the scope to public health-related services contracted by a single city agency, the Chicago Department of Public Health (CDPH). However, our analysis could easily be applied to contracts data across human services departments and jurisdictions if complete data becomes available.

Data on contracted human services have rarely been used for purposes other than financial management of contracts (for exceptions, see [4, 6]. Our aim has been to use these data to provide researchers, health planners, and contract managers with a prototypical example of how to gain a bigger picture of spatial access to human services by funding amount. We apply a spatial perspective to allow stakeholders to see and assess their program’s service allocation decisions in the larger spatial context of their department’s overall service contracting – and thus begin to close department-wide spatial access gaps beyond program-specific gaps.

## Literature

In this section, we first introduce some key research on human service contracting, followed by a summary of selected research on service access and equity mapping.

The U.S. welfare state has long been constituted as a public-private partnership. Aspects of the nation’s history, including federalism, the power of interest groups, and a long-standing preference for a small state bureaucracy have produced, sustained, and expanded this arrangement for nearly two centuries [7–9]. While income transfers -- such as Social Security, Supplemental Security Income, or Temporary Assistance to Needy Families -- may be the most visible form of present-day public welfare provision, public spending on social and health services is a critical part of the social benefit package, especially for low-income people. Indeed, as Allard [10] documents, public expenditures on human services for the poor far exceed public spending on means-tested income transfers. As cited above, recent estimates show that, together, local, state and federal governments spend about a trillion dollars annually on human services [1, 2].

For some time now, the large majority of publicly funded social services have been provided by private, mostly nonprofit, organizations under contract with the government [11–14].^1^ Individuals in need of these services access them via mechanisms very different from those that give access to income transfers: whereas income transfers are sent directly to individuals via direct deposit, a check, or an electronic benefits card, social and health services must be accessed at a particular location, from a specific provider [4]. Similarly, government spending on human services must be allocated to specific providers, which then use those resources to serve clients. Since the 1980s, the federal government increasingly has devolved spending decisions to states and localities [15–21]. For human services spending, this means that while much of the money comes from the federal government, most decisions about which providers will receive contracts to deliver services lie in the hands of state and local officials [4, 6]. As such, data on these contract allocations must be collected from state and local governments. Ethnographic work by [22]; 2007) has illustrated the competitive and political nature of the system by which city, county, and state governments allocate contracts to private human service providers.

A mostly separate body of research addresses questions of access to services and other resources, a classic research problem. Extensive research highlights aspatial and spatial access impediments, including service availability (e.g. number of nearby providers), affordability, acceptability (e.g. multi-lingual services), accommodation (such as hours of operation) and spatial accessibility (travel time or distance) [23–25]. Our focus in this article is restricted to spatial access to services (by funding amount). The question of where areas with a relative under-supply of amenities are located has been addressed most extensively in regards to health services, especially primary care [26–30] and, more recently, healthy food access [31, 32].

Typically (also in our case), spatial access represents “potential access” based on travel times between origin and destination points [24, 33]. A common assumption in measuring spatial access to services is that residents who live close to a service are most likely to access this service -- for example, Allard [10] found that this is true for two thirds of the human service providers he analyzed. Comprehensive data on service utilization, including on the actual times that people travel to a provider (so-called realized access) is hard to obtain city-wide.

Spatial accessibility measures can indicate which neighborhoods are close to service providers but they cannot provide normative guidance on which neighborhoods should be closest to providers. To determine this requires a choice among different notions of equity. Four classic ways to measure equity include definitions that are 1) equality-based (each area receives the same share), 2) need-based (“unequal treatment of unequals”: allocation of public benefits according to need), 3) demand-based (distribution based on economic use or political advocacy), and 4) market-based (ability to pay) [34, 35]. The literature and practice of “equity mapping” combines a focus on equity and spatial accessibility by assessing the spatial equity of public resource distribution [35–37]. Since CDPH seeks to reduce existing inequities in health and related areas [38], we based this analysis on a need-based definition of equity.

## Methodology^2^

The question is whether public health service funding goes to where needs are concentrated. One way to address this is to take geographic areas, such as wards or zip codes, and classify them in two ways: 1) how much funding they get, and 2) how high their needs are. That way each area can be characterized in terms of these two dimensions, e.g. to identify areas with higher funding and higher need, or with lower funding and higher need (Figure 4). This classification can then help target areas for future funding. How needs are defined is addressed in the following Data section.

**Fig. 1:**
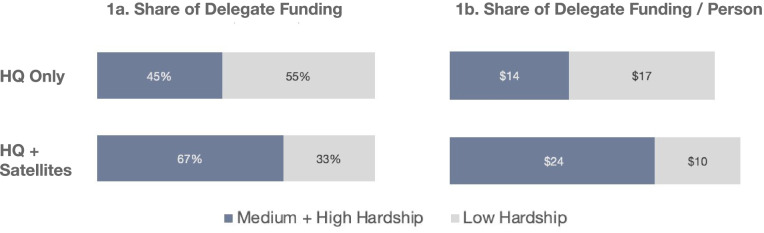
Share of Delegate Funding (Container Approach for Chicago Census Tracts): Headquarters (HQ) only vs. Headquarters (HQ) and Satellite Offices

**Fig. 2:**
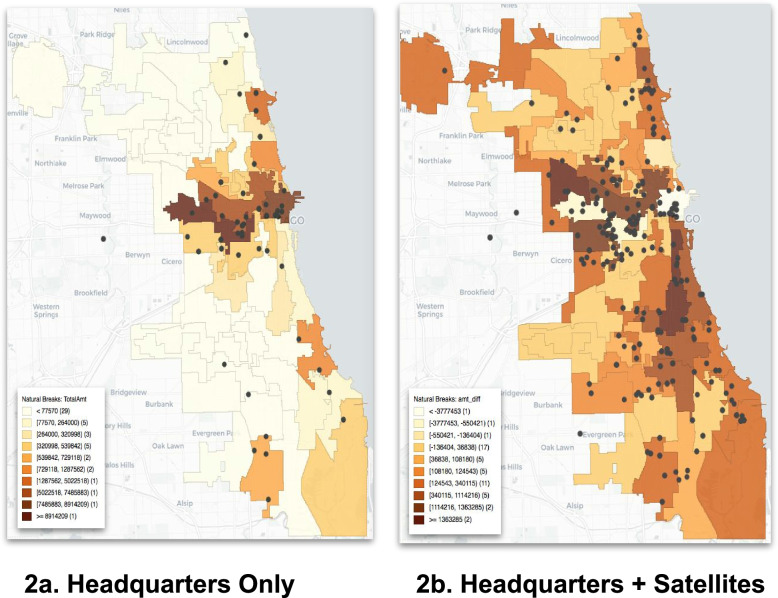
Maps of Delegate Funding (Container Approach for Chicago Wards) (These maps were generated by the authors using GeoDa)

A key prerequisite for being able to measure how much funding an area is receiving is knowing where services are actually delivered -- this requires adding the location of satellite offices to that of headquarters. Since the provider address that is typically recorded in open contracts portals refers to the location of the provider’s headquarters, we compare funding results per area for headquarters versus new data from CDPH on providers’ satellite service site locations. We demonstrate that it is misleading to only use headquarter locations without satellite service delivery locations (Figs. 1 and 2).

There are two classic ways to measure how much funding an area gets: 1) A so-called container approach and a spatial accessibility approach. The container approach indicates how much funding is spent within the boundaries of an area [39–41]. It is often used by default in reports for public consumption [42], as it is easy to compute and communicate. It simply sums funding to service providers within given areas such as wards or community areas. In this article, we also standardize this sum of funding by population (within Chicago Census tracts) to account for varying population sizes in tracts (Fig. 1b). Then the share of funding in areas with lower versus higher needs is compared (Fig. 1a and b).

**Fig. 3:**
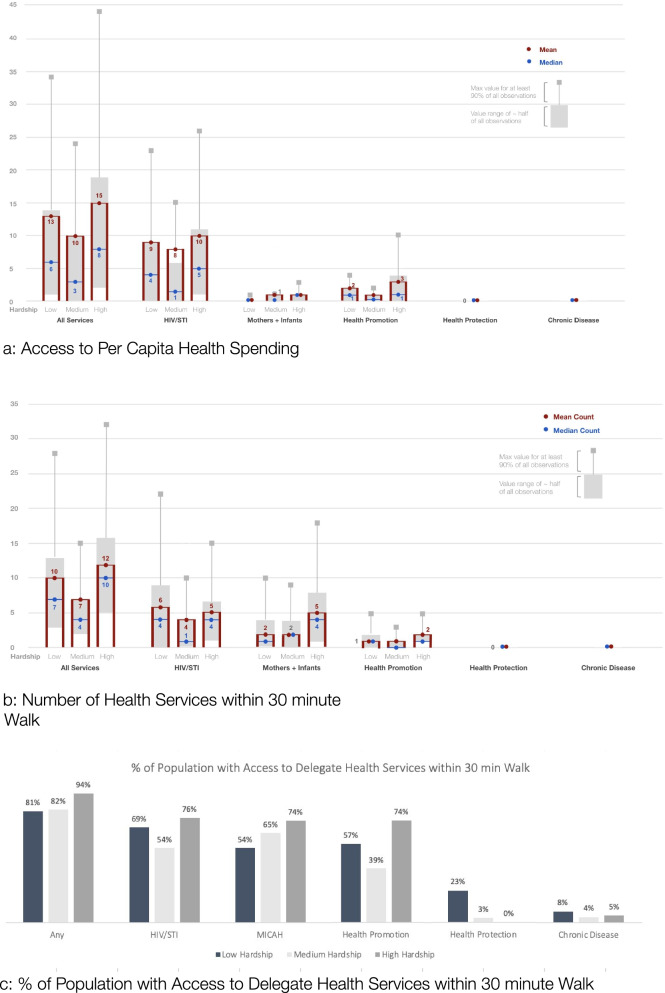
**a**: Access to Per Capita Health Spending. **b**: Number of Health Services within 30 min Walk. **c**: % of Population with Access to Delegate Health Services within 30 min Walk

**Fig. 4:**
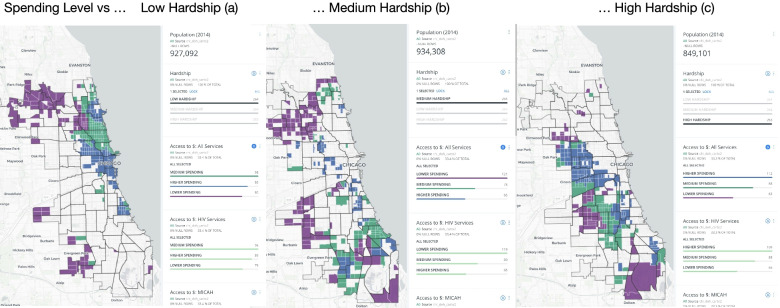
Hardship and Spending Level Maps (These maps were generated by the authors using Carto)

**Fig. 5:**
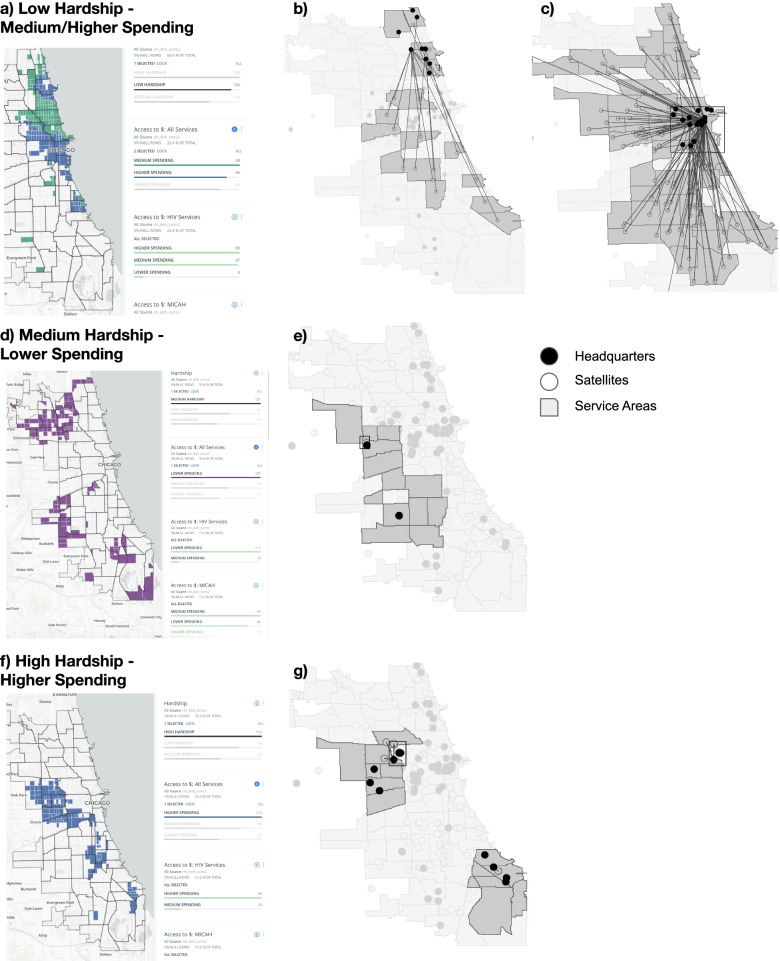
Hardship and Spending Levels Compared to Service Areas. These maps were generated by the authors using Carto and GeoDa

The disadvantage of the container approach is that clients typically do not consider administrative boundaries (like Census tracts) in their decision of which services to access – instead, criteria such as spatial proximity are more relevant [10, 43]. To address this problem, measures of spatial accessibility are often used to assess how close the locations of potential service users are to service providers (e.g., within a 30-min walk). We initially apply the container approach (Figs. 1 and 2) and then extend the analysis to spatial accessibility metrics (Figs. 3, 4 and 5).

To measure spatial access, we compute three standard accessibility metrics: The first two are not related to funding but assess the proximity to health services, namely 1) the time it takes to walk to the nearest health service, and 2) the number of health services within a 30-minute walk (assuming walking speeds of three miles/hour). The third measure, the so-called 2-Step Floating Catchment Area (2SFCA), takes both funding for and spatial access to health services into account [27, 44, 45].^3^ Specifically, it combines coverage measures (how much funding is within reach of potential clients) with spatial accessibility measures (how long it takes clients to reach one or more health service destinations from their point of origin). This is done in two steps. First, starting with the provider, the contract funding amount to a provider is divided by the number of people who can reach the provider within a 30-minute walk – this generates a ratio of funding to nearby population. Second, moving to the point of client origin (in this case, each housing block in Chicago), this ratio is summed within a walking time of 30 minutes between the block centroid and all providers within this walkshed. The resulting funding per capita amount indicates the service dollars that are within walkable reach of residents in a housing block – as opposed to located within the same administrative boundaries, as in the container approach.^4^

The notation for these two steps is as follows (see [47] for details). First, the supply-demand ratio is calculated for each supply point R_j_ (i.e. each health service provider), as shown in Eq. 1:

1 $${R}_j=\frac{S_j}{\sum_i{D}_iI\left({t}_{ij}<T\right)},$$

where S_j_ measures the size of the supply (e.g., the amount of funding). D is the demand at point *i* and I (t_ij_< T) is an indicator function defining the catchment area: It selects only those supply points (S_j_) that are within the catchment area (T) for location *i*.

Second, the supply ratios within the catchment area of each demand point from Eq. 1 are summed into a spatial access measure, as indicated in Eq. 2:

2 $${A}_i^{2 sfca}=\sum \limits_j{R}_jI\left({t}_{ij}<T\right).$$

where A is the 2SFCA access measure for each location *i* (i.e. the point of origin for clients like the block centroid). The rest is as defined above.

We generated the three spatial measures (2SFCA, time to nearest service, and nearby service count) for each health service type and each of the 46,265 housing blocks in the city of Chicago, as blocks represent the smallest spatial Census unit and thus have the greatest small-scale accuracy.^5^ Next we averaged each of the two accessibility metrics (times and counts) and the 2SFCA measure at the Census tract level since our needs data are only available for tracts. Finally, we averaged all three metrics within each category of need, as measured by low, medium and high hardship (detailed below). In addition, each access measure is categorized into one of three groups (e.g. access to lower, medium, and higher spending for 2SFCA). This way each tract could be classified in terms of these three need categories and the three access measures (Fig. 4). Walking times were computed between the centroids of all housing blocks in Chicago following the street network. We developed a new scalable and publicly available Python package to efficiently compute walking and driving times, as well as spatial access metrics [46]. With this package we were able to compute the 2.1 billion travel time pairs for the 46,265 blocks in under 15 minutes.

## Data

In order to address the question whether government contracted service funding goes to where services are needed, several underlying questions have to be addressed, such as 1) how need is defined, 2) where services are delivered, 3) how contract amounts are divided between service delivery sites, 4) where service areas are, and 5) how services are classified. Thus this analysis requires two data inputs: data on where needs are concentrated – the “demand side” – and data on where government contracted human services are provided – the “supply side.” We start with an overview of the data that proxies for human service need and conclude with a discussion of newly collected data from the Chicago Department of Public Health used to illustrate our prototype analysis.

### The potential need for human services

One approach to proxy need is to estimate the number of residents in a neighborhood that might require different types of services, e.g., residents at higher risk of contracting HIV as the target population for HIV health services. Health departments often collect such data for internal analyses and could thus calculate indicators of need that match service types more directly.

In our example we focus on a more general definition of hardship because accurate data for health outcomes are not publicly available for Chicago for small areas such as Census tracts. As a proxy for who potentially needs human services, we draw on a multivariate index called the Hardship Index that is compiled and used by the Chicago Department of Public Health’s Epidemiology and Public Health Informatics program [48]. The index combines the following six socio-economic indicators of public health significance from the U.S. Census Bureau’s American Community Survey’s 5-year estimates (2006-2010): crowded housing, poverty, unemployment, low education, dependents, and per capita income.

The hardship index ranges from 6.7 to 75.1 with a mean and median of about 39 for the 791 tracts in the city of Chicago. Following CDPH practice, we divide the index into three groups of equal size to obtain categories of low, medium and high hardship. Since Chicago’s residential patterns are highly segregated, these groups also tend to be spatially clustered.

### CDPH’s new health service contracts data

Satellite service site locations (where services are actually provided and accessed) are needed to accurately address the question of whether service funding goes to where service needs are. Open contracts data for human services (city, county and state for multiple years) are missing information on these locations of service sites beyond headquarters, as well as other needed data points. Hence, we were unable to use these data for this analysis (see [49]) for a discussion of these data problems). Instead, we worked with the Chicago Department of Health (CDPH) to collect new data in 2018 that included the satellite locations and some other missing features. These data were collected from CDPH’s contracted service providers (called “delegate agencies” by the City of Chicago) with a new service site data form as part of the required contracting documents.^6^ Unfortunately, additional years are not yet available in machine-readable format for a longitudinal study.

The CDPH contracts data contain these key fields: name of the service provider receiving the contract, ID to link to other contract documents, the amount of the contract (in dollars), the start and end dates of the contract, CDPH program name, primary office address, and, via the new satellite service site form, service delivery address(es), contract share per site, and community areas served. CDHP’s 2018 data included 146 contracts to delegate agencies totaling $48.8 million. We focus on the subset of 98 delegate agencies that provide services within the City of Chicago (86% of all 146 delegates) and that offer services at specific sites (69% of all contracts; 362 service sites) instead of citywide. Services can be delivered at the headquarter only, at both headquarters and satellite offices or only at satellite offices. Of the 98 contracts to delegates with satellites that we analyze (totaling $30.4M in contract funding), 21% of services ($6.3M) were provided at headquarter locations only. The remaining 79% of services were delivered at headquarter and/or satellite locations ($21.4M). If site-specific shares were not specified, we divided the total amount equally among all service sites and the headquarter.

We classified the public health service contracts based on CDPH administrative units, including (in the order of contract funding amount) HIV/STI, Health Promotion (e.g. substance misuse prevention and recovery, violence prevention, primary care, and mental health); Maternal, Infant, Child, Adolescent Health (MICAH), including Women, Infants and Children (WIC) and adolescent STI screening and education; Health Protection (eg., lead poisoning prevention and healthy homes, immunization and viral hepatitis), and Chronic Disease. Of the $30.4M in CDPH contracts to delegates with specific service sites (out of $48.8M), 70% are related to HIV (with a small share for sexually transmitted diseases), 16% of funding goes to health promotion, including supporting primary care at federally qualified health clinics (FQHCs) and violence prevention funding, 6% for MICAH, including WIC and adolescent health programs, 5% for health protection and 3% for chronic disease.

## Results

### A prototype spatial access analysis for health services contract data

The methodology and results are part of a prototypical analysis that could be easily extended to research with a bigger picture perspective once contract data become available across jurisdictions (such as city, county and state levels) and across departments (such as social services, health, and housing). Data requirements and standards would be the same across jurisdictions and departments. For the results described in this section, the only difference would be additional data points and categories, which would be straightforward to accommodate. Because it is not yet possible to assess the generalizability of the research results across cities or time, the aim of this article is instead to offer a research methodology that can be generalized to more comprehensive data and other places once these data become available in the future.

To summarize the results upfront, we find the following: 1) The commonly applied practice of summing funding amounts for headquarters locations in areas of interest (like wards or community areas) underestimates the share of funding to higher hardship areas. 2) In general, areas with more hardship are within reach of larger amounts of CDPH contract funding (as well as more health services and shorter time to the nearest service), followed by low hardship areas. Medium hardship areas have comparatively worse access in Chicago. 3) We introduce examples of interactive maps from our spatial decision support system to explore where service access gaps are located. 4) Contextualizing these results by providers’ service area reveals that delegates with headquarters in more advantaged downtown and Northside neighborhoods deliver services to a much broader range of areas across the city than delegates in high and medium hardship areas, which are more locally focused in their service provision. We present the detailed results in turn.

#### Typical headquarter analysis underestimates funding to high hardship areas

We start with the common container method for addressing the question of how much contract funding is going to a particular area like a ward [41, 42]. This method sums the contract amounts for all providers whose headquarters fall within the boundaries of such an area, which is easy to implement and explain. Figures 1 and 2 contain the results for CDPH 2018 contracts to health service delegates (at the Census tract level): The top rows in Fig. 1a and b show that a larger share of funding goes to low hardship areas if only headquarters locations are taken into account. In this case, 55% of delegate funding goes to such areas vs. 45% to medium and high hardship areas. Similarly, $17 per person goes to areas of low hardship compared to $14 per person to areas of medium and high hardship.

Figure 2a explains these results: Headquarters predominate in the more affluent downtown and Northside areas. However, the bottom row of Figs. 1 and 2 illustrate why this common way of addressing our question is misleading. After adding the satellite service site locations from CDPH’s service site form, the results are reversed because service sites predominate in medium and high hardship areas. Therefore, the majority of contract funding now no longer goes to areas of low hardship but to those with medium and high hardship. Specifically, the medium/high hardship share increases from 45% to 67% (Fig. 1a) and from $14/person to $24/person (3b) when service sites are taken into account. Figure 2b confirms the much broader spatial reach of contract funding when service sites are taken into account.

#### Contracted health services and funding are more accessible to high hardship areas

As discussed above, the container approach ignores the fact that clients tend to not choose services based on administrative boundaries. An alternative approach is to estimate spatial access to services based on walking times to services. Figure 3a-c summarize the results for the three spatial accessibility measures discussed in the methods section. These results are broken out for the three groups of hardship and the different types of health services described previously. Figure 3a is based on the Two-Step Floating Catchment Area method, which measures access within a 30-min walk of a block’s center to health funding per person near providers. Another way to assess spatial access is through the number of services reachable on a 30-min walk (Fig. 3b) and the share of the population who can reach at least one health service within a 30-min walk (Fig. 3c).

As before, the purpose of these figures is to address the question whether government funding for services goes to where the need is for those services. It turns out that high hardship areas do have the highest health service access in Chicago, which is consistent with the health department’s equity goals. Interestingly, the neighborhoods with the next best level of spatial access are low hardship areas while medium hardship areas tend to trail both. (Figures 3a-c and Table S1).

For example, for all services taken together, high hardship tracts have access to an average of $15 per person near the service provider compared to $13 in low hardship tracts and $10 in medium hardship tracts (Fig. 3a). The equivalent median values are $8, $6 and $3. Although the range of funding for about half of all tracts in high hardship areas overlaps most with those in low hardship areas, the high hardship upper value range and upper limit still exceed that of the low hardship areas. This pattern generally also holds for the number of health services within a 30-minute walk (Fig. 3b) and the share of population with access to services within the same travel time (Fig. 3c). One exception is access to the number of HIV/STI services, which is highest in low hardship areas – reflecting more clustered services on the low-hardship Northside.

Another exception are health protection and chronic disease (breast health) services, which are also higher in low hardship areas. However, at closer inspection these services are not a good fit for spatial access metrics for the following reasons. The majority of health protection funding comes from the Hospital Preparedness Program to prepare hospitals for emergency and disaster response. Two of the three contracts went to hospitals and are not classical health services that clients can access. In addition, CDPH’s health protection bureau includes city-staffed services such as CDPH immunization clinics, lead inspectors, and environmental protection, which are not reflected in human service contracts. Similarly, the nine chronic disease contracts for breast health primarily went to institutions in the medical complex in the Illinois Medical District, West of Chicago’s loop, which have larger service areas than smaller nonprofit providers.

Figure 3a-c also demonstrate that spatial access to per capita health spending (Fig. 3a) generally increases with funding for services -- with highest to lowest funding from HIV/STI to health promotion, mothers and infants, health protection and chronic disease. However, when only the number of delegates are taken into account (not the contract amount), as in Fig. 3b and c, services for mothers and infants are offered through more providers than health promotion services (28 vs. 9 service sites) and are thus more accessible. Since funding levels for health protection and chronic disease are lower, there are fewer providers and thus lower spatial access for these services. However, for both services, spatial access is best in low hardship areas, reversing the general pattern in these cases (see discussion below).

In summary, a larger share of CDPH contract funding for health services does seem to go to where needs are highest in Chicago. The finding that areas with medium hardship have proportionately less access could be explored in more detail by contract managers and health planners. In the next section, we examine these findings more closely, drawing on the interactive maps we built for this project and the results about providers’ service areas from CDPH’s 2018 data.

## Discussion

### Interactive exploration of spatial access gaps of health services

A series of new interactive maps enable the exploration of these results at the tract and block level using Carto’s web-based platform. These maps can be used as 1) a diagnostic tool to identify data problems and missing data; 2) as a tool to save CDPH staff time in answering frequent questions about how much contract funding goes to political districts and other areas; and 3) as a prototype to demonstrate and assess the usefulness of different data summaries for contract managers, health planners and other staff.

Figure S1 displays one example of these interactive maps, a Census tract map of access to per capita spending for all services – additional layers of service sites can be displayed. Pop-up views contain results for each tract and service site. Figure 4 illustrates how contract managers or health planners could interactively explore this map by filtering hardship and spending levels. For instance, in Fig. 4a they could select low hardship areas and color tracts by level of access to spending. Figure 4b and c show the same for medium and high hardship areas. Not surprisingly, tracts closer to the periphery of the city have lower service access.

### Spatial accessibility results vs. service areas

Finally, Figure 5 explores why spatial access was found to be higher in low hardship than medium hardship areas (see Table S1 and Fig. 3a-c). The figure combines the interactive maps with data from CDPH’s service site form about community areas that are served by delegates. Figure 5a highlights the low hardship tracts in Chicago, which include the wealthier Northside and downtown areas. Figure 5b selects some of the delegate headquarters (filled black circles) in the Northside to identify the associated service sites (outlined circles) and service areas (grey areas) of these headquarters. It turns out that these service areas include many community areas on the poorer West and South sides. This pattern is even stronger for headquarters downtown with service sites all over the city (Fig. 5c). One thing to note is that we divided contract amounts equally between headquarters and satellite offices (Fig. 5b and c). If contract-funded services are not actually delivered at headquarters locations, we are over-allocating contract funds to low hardship areas with many headquarters locations (service delivery at headquarters could only be identified as of 2019 in CDPH’s data).

In contrast to this widespread service delivery pattern of delegates with headquarters on the Northside and in downtown Chicago, headquarters in high hardship areas with higher spending (Fig. 5f) deliver services much closer to their offices -- as shown for a subset of delegates in Chicago’s West- and Southside. Lastly, Fig. 5e selects two delegates near medium hardship areas with lower spending (Fig. 5d) to see if their service areas cover some of the peripheral “spatial access gap” tracts: Indeed, they do (Fig. 5e). This analysis illustrates that the initial spatial accessibility results should be supplemented by additional interactive exploration of the results to better understand the underlying dynamics and avoid identifying false access gaps.

## Conclusion

These findings demonstrated the feasibility of applying classic spatial access metrics to new administrative contracts data in order to add a financial dimension to the typical spatial access gap analysis. With these results, health planners can supplement their standard needs assessments with service funding data to identify areas of concentrated poverty and low access to contract-funded services – or include access to human service funding in the design and evaluation of place-based initiatives (in our case, the *Elevated Chicago* target areas). We also illustrated that the typical approach, which sums the amounts going to headquarters within area boundaries, is misleading and will underestimate funding to medium and high hardship areas. This is relevant for results shared with aldermen, advocates or service providers who want to know how much services funding is going to their wards or community areas.

Finally, while CDPH’s service site form closes some of the gaps in open contracts data, other gaps remain – crucially, data about intermediaries is still missing. Another remaining challenge is that these data do not cover all funding sources. They do not include government funding that flows into city neighborhoods through sources such as Medicaid payments, payments to clients for services, foundation funding, or human services provided directly by city staff. Additionally, services funded by other departments (such as social services or housing) or other jurisdictions (such as the county or state) could not be included from open sources because too much information is still missing [49]. We caution that what might look like a service desert in our spatial access analysis might not actually be one if these other sources were taken into account. Nevertheless we hope that this work inspires the closing of these remaining data gaps and supports the wider integration of research on spatial accessibility and human services contracting.

## Supplementary Information

**Additional file 1: Figure S1.** Spatial Decision Support System. **Table S1.** Hardship and Spending Levels.

## Data Availability

The main dataset generated and analyzed during the current study is not publicly available as it belongs to the Chicago Department of Public Health (CDPH). Please contact CDPH as part of a reasonable request for public research. The publicly available data (IRS 990) can be obtained from the corresponding author.

## Abbreviations

2SFCA: 2-Step Floating Catchment Area
CDPH: Chicago Department of Public Health
FQHC: Federally Qualified Health Clinic HQ Headquarter
MICAH: Maternal, Infant, Child, and Adolescent Health
STI: Sexually Transmitted Diseases
WIC: Women, Infants and Children

## Footnotes

1 It is notable that in some areas of social service provision, particularly workforce development and health care, the share of private, for-profit providers is rising.

2 We conducted a 2-day “design sprint” in Chicago with interviews with Chicago government officials responsible for contracting decisions and health policy to inform our methodology and data collection, and to make the results relevant to human service stakeholders. The sprint is documented visually here: https://bit.ly/36W3MUG.

3 There are several extensions that we do not consider in this analysis ([28, 30]; Saxon and Snow, 2019).

4 We share pre-computed travel times between tracts within a 100 km range for these three travel modes at a national level and for tracts and blocks in the largest 20 US cities. These travel times can be used to calculate accessibility metrics in packages like Noel [46] and Saxon et al. [47]. Documentation for Noel [46] can be accessed here: https://bit.ly/37NyWNw.

5 This approach avoids inaccuracies in spatial access metrics that are calculated for larger spatial units as shown in Jankowski and Brown [31].

6 Available for download at: https://bit.ly/37Sm2O8
