# Supplementary material for: Does health service funding go where the need is? A prototype spatial access analysis for new urban contracts data
Source: BMC Health Serv Res. 2022 Jan 9;22:45. doi: 10.1186/s12913-021-07370-8 (PMC8744308; doi:10.1186/s12913-021-07370-8)
Supplement: Supplementary file 1 — Additional file 1: Figure S1. Spatial Decision Support System. Table S1. Hardship and Spending Levels. [file 12913_2021_7370_MOESM1_ESM.pdf]

Supplementary Materials

Explanation of Figures 3a-c:

Figures 3a and 3b show the typical mean values in the context of the variation around the mean. Because the distribution of service funding is skewed upwards, i.e. some areas are receiving larger shares of funding than others, a comparison of the distribution across hardship categories helps gauge how much high hardship values differ from the lower hardship categories. Mean values are shown in red in Figures 3a and 3b. To reflect the skewed distributions, the generally lower median values are added as well in blue, with two measures of variation borrowed from boxplots: the grey rectangle depicts the range of values of (in this case) about half of all observations and the grey square marks the upper limit or hinge for (in this case) 90% of observations. The grey rectangle represents the range of values within the 25th and 75th percentile of sorted values while values beyond the grey square are classified as outliers (with a hinge of 1.5). Since Figure 3c is based on percentages rather than mean values, it does not include measures of variation.

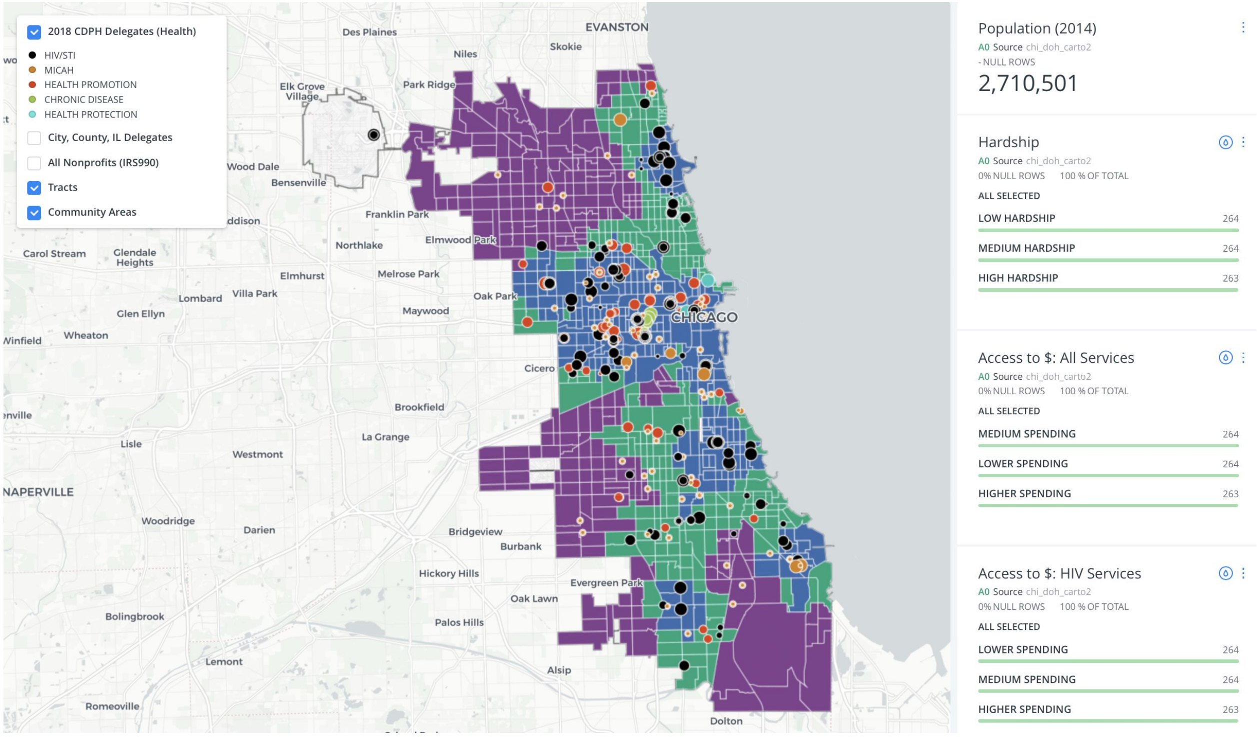

Figure S1: Spatial Decision Support System

a) Hardship and Spending Levels by Tracts (#)

	High Hardship	Medium Hardship	Low Hardship
Higher Spending	113	64	87
Medium Spending	88	78	97
Lower Spending	63	121	80
Total Tracts	791		

b) Hardship and Spending Levels by **Tracts (%)**

	High Hardship	Medium Hardship	Low Hardship
Higher Spending	**14%**	8%	11%
Medium Spending	11%	10%	**12%**
Lower Spending	8%	**15%**	10%

c) Hardship and Spending Levels by Population (#)

	High Hardship	Medium Hardship	Low Hardship
Higher Spending	312,418	167,478	299,550
Medium Spending	262,031	270,587	319,187
Lower Spending	274,652	496,243	308,355
Total Population	2,710,501		

d) Hardship and Spending Levels by **Population (%)**

	High Hardship	Medium Hardship	Low Hardship
Higher Spending	**12%**	6%	11%
Medium Spending	10%	10%	**12%**
Lower Spending	10%	**18%**	11%

Table S1: Hardship and Spending Levels

Table S1b is a 3x3 table to indicate the share of tracts in each of the three hardship categories vs. the three spending levels (i.e. where lower, medium and higher spending all have the same numbers of tracts). Table S1d is structured like Table S1b but for people instead of tracts. Since there are nine cells, an equal share would be about 11%. The three categories above this threshold in both tables are medium hardship areas with lower spending, high hardship areas with higher spending, and low hardship areas with medium spending.

Links to resources by the Center for Spatial Data Science referenced in the article:

1. A new Python package “access” released in July 2020 as part of the PySAL library
2. Pre-computed travel time matrices by modes for U.S. neighborhoods can be downloaded.
3. 1 and 2 are available at https://access.readthedocs.io/en/latest/
4. For background: Saxon, James, Julia Koschinsky, Karina Acosta, Vidal Anguiano, Luc Anselin, and Sergio Rey. (2021). An Open Software Environment to Make Spatial Access Metrics More Accessible. Center for Spatial Data Science, University of Chicago Working Paper (under review). Preprint available at https://www.researchgate.net/publication/350338824_An_Open_Software_Environment_to_Make_Spatial_Access_Metrics_More_Accessible

5. Logan Noel developed an additional Python access package to compute travel times (walking and driving) and access metrics efficiently at scale in Python: https://pypi.org/project/spatial-access/
6. Documentation for the package available at: https://bit.ly/37NyWNw

7. Visual summary of design sprint in 2017: https://bit.ly/36W3MUG
